# Capturing sexual contact patterns in modelling the spread of sexually transmitted infections: Evidence using Natsal-3

**DOI:** 10.1371/journal.pone.0206501

**Published:** 2018-11-01

**Authors:** Samik Datta, Catherine H. Mercer, Matt J. Keeling

**Affiliations:** 1 Zeeman Institute: SBIDER, Warwick Mathematics Institute and School of Life Sciences, The University of Warwick, Coventry, CV4 8UW, United Kingdom; 2 National Institute of Water and Atmospheric Research, Evans Bay Parade, Wellington 6021, New Zealand; 3 UCL Centre for Population Research in Sexual Health and HIV, Institute for Global Health, University College London, London, WC1E 6JB, United Kingdom; University of Waterloo, CANADA

## Abstract

**Background:**

Understanding the spread of sexually transmitted infections (STIs) in a population is of great importance to the planning and delivery of health services globally. The worldwide rise of HIV since the 1980’s, and the recent increase in common STIs (including HPV and Chlamydia) in many countries, means that there is an urgent need to understand transmission dynamics in order to better predict the spread of such infections in the population. Unlike many other infections which can be captured by assumptions of random mixing, STI transmission is intimately linked to the number and pattern of sexual contacts. In fact, it is the huge variation in the number of new sexual partners that gives rise to the extremes of risk within populations which need to be captured in predictive models of STI transmission. Such models are vital in providing the necessary scientific evidence to determine whether a range of controls (from education to screening to vaccination) are cost-effective.

**Method and results:**

We use probability sample survey data from Britain’s third National Survey of Sexual Attitudes and Lifestyles (Natsal-3) to determine robust distributions for the rate of new partnerships that involve condomless sex and can therefore facilitate the spread of STIs. Different distributions are defined depending on four individual-level characteristics: age, sex, sexual orientation, and previous sexual experience. As individual behaviour patterns can change (e.g. by remaining in a monogamous relationship for a long period) we allow risk-percentiles to be randomly redrawn, to capture longer term behaviour as measured by Natsal-3. We demonstrate how this model formulation interacts with the transmission of infection by constructing an individual-based SIS-P (Susceptible—Infected—Susceptible—Protected) transmission model for the spread of a generic STI, and observing overall population demographics when varying the transmission probability within a partnership, recovery rate and the level of population protection (e.g. from vaccination where applicable).

## Introduction

Sexually transmitted infections (STIs) affect many hundreds of millions of people globally every year [1]. HIV has been on the rise since the 1980s, and other infections such as gonorrhoea, chlamydia and the herpes simplex virus (HSV) are present and increasing in many countries; the vast majority of people are infected with one or more strains of the human papillomavirus (HPV) in their lifetimes [2]. The annual healthcare costs of STIs in the USA alone has been placed at over $15 billion [3]. For HPV, the world’s most common STI, vaccination programs exist across the world, including: the UK [4, 5], the Netherlands [6], and many others around the world (e.g. [7]).

Modelling the spread of STIs needs to account for multiple rich heterogeneities in individual-level characteristics. The spread of other transmissible infections such as flu, measles or pneumococcal infections, which are airborne or spread through fomites, are often successfully captured by assuming random (homogeneous) mixing within the population [8–10]. Alternative assumptions that replicate more of the biology include: household models which account for greater transmission risk within family groups [11, 12], age-based mixing patterns which account for different behaviour [13–15] and to a lesser extent the patterns of social connections [16, 17]. A recent analysis combined data from the contact survey POLYMOD [14], time use data and serological data from Italy to produce a mixing matrix for frequency and duration of contacts [18]. In contrast, human sexual behaviour depends strongly upon several compounding factors, including age, sex, sexual preference and social and cultural norms [19], as well as underlying heterogeneity in the number of sexual partners [20–22] and trends across time [23, 24]. There is extreme heterogeneity between individuals in the number of sexual partners [24], partnership networks [25] and scale-free sexual networks [26]; furthermore, trends shift across time [24, 27]. These heterogeneities lead to different STI prevalences, including by gender and age group [28].

Modelling sexual partnerships has historically been performed in a variety of ways. Mimicking methods from non-STIs, some researchers have utilised deterministic ordinary differential equation (ODE) models to simplify the complex interaction between individuals [29]; while other have adopted a more complex formulation, subdividing the population based on characteristics, including: age and sexual activity [30, 31]; or previous sexual experience [32]. However, the majority of available sexual behaviour data are not of sufficient resolution to estimate combined factors of age preference, partnership duration, and changing behaviour with age.

Unsurprisingly, there have been intense efforts to try to understand the sexual mixing patterns in populations, either focusing on eliciting all the links within one population [33] or gaining a generic understanding of patterns within the community [34]. Britain’s National Surveys of Sexual Attitudes and Lifestyles (Natsal) are among the largest and most comprehensive studies of sexual behaviours in the world [24]. Three surveys have been carried out to date, approximately decennially, in 1990-1991, 1999-2001 and most recently in 2010-2012. The detailed data from each respondent include: information on demographic characteristics; the number of partners over a range of time periods (from days up to years); and many other aspects of sexual behaviours. We use data from the Natsal-3 survey (sampled over 2010-12) to determine distributions for the rate of new partnerships that involve unprotected sex and can therefore allow the spread of STIs. In particular, we show that the heterogeneity reported in the Natsal-3 survey can be captured by parsimonious distributions of stochastic annual rates—leading to a mixture of Poisson distributions over time. These stochastic rate distributions depend on four personal characteristics: age (years), sex (male/female), sexual orientation (heterosexual/any other) and previous sexual experience (yes/no). Individuals in the population are given a risk-percentile which determines the value extracted from the distribution of rates, with the distribution used matching the individual’s characteristics (e.g. age, sex, etc). This means that high risk-percentile individuals will consistently have high rates of new partners relative to their peers. However, we know that individual behaviour patterns can change (e.g by starting or ending a long-term relationship), we therefore allow risk-percentiles to be randomly redrawn with a low probability—this allows us to capture longer term (5-year and lifetime) patterns reported in Natsal-3.

As a first use of the partnership characteristics derived from Natsal-3, we construct a simple individual-based model for STI transmission, and observe patterns of disease prevalence when altering the probability of transmission upon entering a new partnership, the recovery rate from infection, and the level of protection for new individuals entering the population. This last factor could be due to several reasons: more widespread education about protective sex, including promoting better use of condoms, higher availability of condoms, or level of vaccination (for STIs such as HPV and hepatitis B). We refer to this as “probability of protection” for the remainder of the paper.

## Methods

Here we describe the formulation of the transmission model as two distinct processes: the formation of new partnerships; and the probabilistic transmission of infection within the new partnership. Note that, although information on concurrent partnerships was available from Natsal-3, the data were not of high enough resolution to use, and hence we consider each partnership chronologically separate. This is a necessary assumption in the model framework, and is discussed in greater detail in the Discussion.

For the purposes of the analysis carried out here we use three separate pieces of data:

1. Number of new sexual partners (where there was unprotected sex) of all respondents over a one-year period, a five-year period and lifetime, together with the age, sex, sexual preference (captured by whether they reported ever having had a same-sex partner or partners) and whether each respondent was a virgin.
2. The age of the three most recent partners and how this relates to the characteristics (sex and age) of respondents using information on the Natsal-3 survey.
3. The age of first sex (and if this was with an opposite-sex or same-sex partner).

We then aggregate data from individual respondents within 5-year age groups (to increase the sample size in each), but differentiate by sex, sexual preference and past sexual experience (i.e. disaggregating virgins from those who have had one or more sexual partners); we use the parameter *c* to refer to these individual-level characteristics including the age of the respondent. In the work that follows we will only focus on new sexual partnerships that involve unprotected sex, but for brevity will often simply refer to these as new (sexual) partnerships. Condomless sex is a necessary assumption in the model framework, and is discussed in greater detail in the Discussion.

### Data

Full methodological details of Britain’s third National Survey of Sexual Attitudes and Lifestyles (Natsal-3) have previously been published, and are available from the study’s website: www.natsal.ac.uk, including the Natsal-3 questionnaire [35, 36]. Briefly, Natsal-3 was a complex multi-staged survey involving a stratified, clustered probability sample design. Altogether, 15,162 interviews with people aged 16-74 years resident in Britain were completed between September 2010 and August 2012. The response rate was 57.7%. Computer-assisted personal interviewing (CAPI) was used, with computer-assisted self-interview (CASI) for the more sensitive questions, including those about the number and gender of sexual partners, and whether condoms were used with the partner, and a detailed question module on participants’ three most recent partners. An anonymised dataset is available to academic researchers from the UK Data Service, https://discover.ukdataservice.ac.uk/; SN: 7799; persistent identifier: 10.5255/UKDA-SN-779-2.

### Defining partnerships

We first consider the annual number of new sexual partners (where there was unprotected sex, defined from the data as condomless sex on at least one occasion), for individuals who are already sexually active. We begin by assuming that the observations can be generated by each individual having a particular stochastic rate, chosen from a distribution, of acquiring a new partner—hence the partnership dynamics are Markovian and thus can be readily simulated. Note that the number of new partnerships, and not partnership duration, is modelled here—this is discussed further in the discussion.

For a given stochastic rate, the expected number of new sexual partners in a year is therefore assumed to be Poisson distributed. Hence, if the observed distribution of new partners over one year is *O*_1_(partners|characteristics) we simply seek a set of parameters $\theta_{i}^{c}$ such that:

$$\begin{array}{c} O_{1}\left(p|c\right)\;\approx \;\sum_{m}\theta_{m}^{c}\;P\left(p|m\right)\;=\;\sum_{m}\theta_{m}^{c}\;\frac{e^{m}m^{p}}{p!} \end{array}$$ (1)

where P(x|m) is the probability of drawing *x* from a Poisson distribution with mean *m*. *θ* (normalised to sum to one) therefore defines the distribution of stochastic rates for each characterised-class *c*; with the values of *θ* determined to minimise the difference between the observed and calculated distributions. Surprisingly, simply using *m* = 0, …, 7 (where *m* is the mean of the Poisson distribution) is sufficient to capture the heterogeneity in the number of partnerships. We achieve this fitting to the annual data using a maximum likelihood approach for a fixed value of *m*, and assessed the best *m* using AIC.

This approach works well for a single year—each individual could choose their stochastic rate from the distribution appropriate for their characteristics. However, over longer timescales this random approach for each year will not work well, repeatedly picking from the distribution would lead to little variation in expected lifetime numbers of partners (as we would be summing across multiple random samples). An alternative solution is to give each individual (at birth) a percentile, which determines the value selected from the appropriate distribution; those with higher percentiles are continually associated with high rates of acquiring new sexual partners (relatively for their age, sex and other characteristics). We therefore define the function *Q*(*P*|*c*) which translates a percentile risk *P* into a stochastic rate of new sexual partners, for a given set of characteristics. Unfortunately, this fixed percentile approach leads to too much population heterogeneity, as it cannot account for the potential change in individual behaviour over time (e.g. entering or leaving a long-term stable relationship).

As a compromise between lifetime risk percentages and random annual risk percentages, we define an annual probability *q* of randomly re-drawing a percentile risk (changing sexual behaviour) which depends on individual characteristics (sex and sexual preference) and is a piece-wise linear function of age. This parameterisation is performed by observing predicted numbers of partners over time scales longer than a year (3 years, 5 years and lifetime) and comparing this to the recorded Natsal-3 data. For example, given data on partnerships over 3 years (which is long enough to illustrate the principles, but not so long that there are too many combinations) we predict the following distribution:

$$\begin{array}{ccc} O_{3}\left(p|c\right) & \approx & {\left(1-q_{c}\right)}^{2}\sum_{m}\theta_{m}^{c}\;P\left(p|3m\right)\\ & & \;+2q_{c}\left(1-q_{c}\right)\sum_{p_{1}+p_{2}=p}(\sum_{m}\theta_{m}\left(c\right)P\left(p_{1}|2m\right))(\sum_{m}\theta_{m}\left(c\right)P\left(p_{2}|m\right))\\ & & \;+q_{c}^{2}\sum_{p_{1}+p_{2}+p_{3}=p}(\sum_{m}\theta_{m}\left(c\right)P\left(p_{1}|m\right))(\sum_{m}\theta_{m}\left(c\right)P\left(p_{2}|m\right))(\sum_{m}\theta_{m}\left(c\right)P\left(p_{3}|m\right)). \end{array}$$ (2)

For simplicity of notation we have assumed that the same characteristics, *c*, hold for each individual over the entire 3-year period, although in practice they will age. The three terms in Eq 3 relate respectively to (i) maintaining the same percentile for all three years, (ii) changing percentile once, and (iii) changing percentile twice so that all three years use different values. This allows individuals to change partnership formation rates rapidly, as is commonly observed, at least at younger ages [24].

We infer the parameters that underpin the partnership formation rates (*θ*) using a maximum likelihood approach, fitting to the annual number of new partners (and new partners without the protection of a condom) in each age-group as given by the Natsal-3 data. Determining the probability of transitions between risk-percentages (*q*) is more complex, Eq (3) shows the number of terms required when predicting the distribution of partnerships over just 3 years, this obviously increases when one considered the number of lifetime partnerships—especially when the age of first partnership also needs to be taken into account. This restricts our ability to use a likelihood based approach for *q*; instead we utilise a simulation-based approach generating a synthetic population that can be compared to the data. We take the function *q* to be a continuous function of age given by a broken-stick model (with 4 free parameters) and use a different set of parameters for each gender. The parameters within the function *q* are inferred through a ABC-type approach [37], minimising the difference between the observed and simulated distributions of partners over different durations (3 years, 5 years and lifetime).

Natsal-3 recruited participants aged 16-74, although for the spread of STIs the bulk of relevant sexual activity occurs by the age of 50 [24, 38] and we use this age as an upper bound in the model. To account for sexual activity below the age of 16, we need to modify our fitting procedure. The stochastic new partnership formation rates for individuals between 10 and 15 are determined using both the information on age of first sex together with the number of partners over longer times (3 years, 5 years and lifetime), with the expected rates increasing non-linearly up to age 16.

For individuals who have engaged (or will engage) in same-sex activity (about 7% of men report ever having had same-sex sexual experience by the age of 45 [24] the formulation is slightly more complex, as both the number of opposite-sex and same-sex partners need to be defined for each percentile. We do this by a similar method to that outlined above, but partition this population into those who only engage in same-sex activity and those who have sex with both men and women. Thus, for each individual we choose the rate of new partnerships by whether or not the individual has engaged or will engage in same-sex activity. If not, partnership rates are shown in the Results section. For those who engage in same-sex activity, the rates and sex of the partner is determined using distributions in the Supplementary Material.

The above detailed model fitting, only determines one part of the network structure—the degree distribution over time. The other component is to determine who has sex with whom. In principle, this is a large matrix that gives the probability that someone with characteristics $\tilde{c}$ and percentile $\tilde{P}$ has sex with an individual with characteristics *c* and percentile *P*. The characteristics of a partner (in terms of age and sex) are recorded within Natsal-3, in addition we choose the percentile of the partner using a configuration model [39], such that the probability that a partnership is made with someone of percentile *P* is given by: *Q*(*P*|*c*)/∑_*p*_ *Q*(*p*|*c*), assuming the characteristics have already been identified. While this is a useful default assumption, and is consistent with the lack of information on the past behaviour of sexual partners, it could be argued that there will be some level of assortativity between partnerships, with low-percentile individuals (looking for long stable relationships) more likely to partner other low-percentile individuals. However, this element of structure cannot be inferred from the available data (as it would require detailed information of the number of partners of each partner); instead the level of assortativity can only be estimated either by matching to the consequences of the partnership network (in terms of the level and distribution of infection) or from having much more detailed information on the entire partnership network (as seen in small detailed studies [33]).

### Defining transmission

The above formulation has determined the number of new (unprotected) sexual partnerships an individual engages with over different periods of time. To translate this to the population scale requires considerable care. Two approaches are feasible. The most intuitive and mechanistic approach would be to explicitly model the full transmission dynamics within partnerships. However, this is fraught with difficulties, most notably that using partnership formation rates calculated above would lead to double-counting of partnerships—as each individual will both generate their own partnerships and be involved in partnerships initiated by others. Hence, to correctly utilise this approach, partnership formation and dissolution at the population level would necessitate a (non-trivial) rescaling of all the parameters defined above. However, if we are prepared to insist that all partnerships occur serially, with no overlapping (concurrent) partnerships, then a simplification becomes possible. Instead of modelling bi-directional transmission within a partnership, we can take an individual-centric approach and only model transmission to an individual every time they form a new partnership; with the rates of partnership formation as specified above. (An alternative way of conceptualising the individual-centric approach is that we model a focal group who pick partners from another (larger) population whose infectious status mirrors that found in the focal group. This ensures that the pattern of partnerships in the focal group matches the Natsal-3 statistics, and that infection is modelled correctly.) These two approaches of rescaled partnership formation rates with bi-directional transmission and an individual-centric methodology with one-directional transmission are identical if concurrent partnerships can be ignored.

We note that our mathematical model makes a number of simplifying assumptions, which greatly reduce the complexity (and dimensionality) of the model and allow us to exploit the heterogeneities captured by the Natsal surveys. The main assumptions are:

- Homogeneous partnerships. We have assumed that all partnerships are associated with equal risk of transmission, however there is considerable heterogeneity between partnerships both in terms of frequency and type of sexual act, which will influence the transmission probability.
- Lack of concurrency. Although our model does not explicitly model concurrent partnerships, it does include such concurrent relationships in the calculation of partnership rates; therefore, the model only fails to capture transmission within the stable partnership due to infection that comes from a concurrent relationship. We believe that inference of the transmission parameter necessary to capture a given level of infection in the population will counteract much of this bias.
- Instantaneous transmission assumption. We have assumed that transmission of infection happens as soon as a partnership begins; this is clearly a simplification but will have a minimal effect on the dynamics as further transmission will not occur until a new partnership has begun. It may however introduce a slight bias in the age dependent level of infection, as individuals will be infected slightly earlier.
- Perfect protection. For simplicity we have assumed that both condoms and vaccination provide complete protection against infection. Both of these simplifications can be overcome by a scaling of either the partnership data (including some partnerships that only have condom protected sex) or the vaccination rate (considering vaccine failure and waning).
- Lack of assortativity. While assortativity appears to be the norm in most social interactions (those with more contacts generally meet others with more contacts), given that the Natsal surveys are individual focused there is no method of obtaining such information from these studies.
- Lack of explicit partnerships. While the lack of explicit partnerships allows our model to be parsimonious, it also provides some restrictions on its use. For example, interventions (such as contact tracing or couple-targeted controls) which are targeted at partnerships are not easily incorporated within our framework. Moreover, information from relationship prevalence data, which is often used to establish transmission probabilities across partnerships, cannot readily be used within our framework.

However, despite these assumptions, we feel that the limitations are more than compensated for by the parsimonious nature of our model.

### Stochastic simulations

We simulate the partnership dynamics and transmission of a generic STI using an individual-based stochastic simulation, taking the stochastic rates and probabilities as given above. A fixed population size of 20,000 individuals is used in simulations, which allows rapid simulation without stochastic fluctuations dominating behaviour; this population size is suitable for the parameter ranges used here, although in practice may need to be increased to simulate STIs with very short infectious periods. There are approximately equal numbers in each age class, which is representative of the British population in the age range considered [40]. Individuals are sampled from the population distribution in Natsal-3, and are aged 10-50 (note that Natsal-3 sampled those from age 16, and we infer distributions for those aged 10-15 using the methods above); and as they age through time during simulations, an individual reaching 51 is immediately replaced by one aged 10—thus keeping the population size constant.

Upon an individual forming a partnership, if the partner is infected, then there is a probability of the individual becoming infected instantaneously. We choose a generic STI rather than focusing on a particular STI such as chlamydia, or a particular type such as bacterial or viral STIs. There are reason for this: (i) to allow for basic conclusions to be drawn from this simple example, and (ii) to allow the model framework to easily be adapted for simulating specific STIs. We assume SIS-P (Susceptible—Infected—Susceptible—Protected) dynamics, which is typical for several STIs (particularly bacterial STIs), meaning that after the natural recovery from an infection (or following treatment) that individual is immediately able to be reinfected. Individuals are also able to be protected from birth, meaning they cannot be infected at any time. Again, considering the time-scales involved with many STIs, this is a reasonable and parsimonious assumption as any recovery period is relatively short. We keep the model simple by assuming a fixed probability of transmission when an infected individual partners with a susceptible individual (*β*, default value 0.7), a fixed recovery rate, the inverse of the infectious period (*γ*, default value 1 per years), and a fixed rate of protection of individuals *ν* as they enter the model, which for simplicity we assume offers lifetime protection at 100% efficacy. We vary *β*, *γ* and *ν* in simulations, to observe the effect of disease prevalence both at the population level and within age groups.

Simulations run for 100 years to allow the system to attain the endemic equilibrium. Throughout we use a discrete time step of Δ*t* = 20 days (hence a stochastic rate *R* is replaced by a probability 1 − exp(−*Rδt*) that an event occurs within the time step); sensitivity analysis has shown that results are insensitive to a time step of this duration.

## Results

### Partnership characteristics

**Fig 1:**
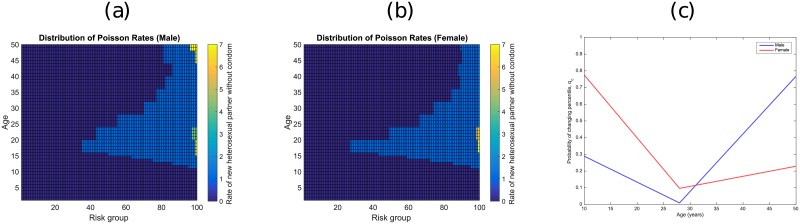
How partnership formation rates change with age. Plots (a) and (b) show distributions for men and women respectively. Plot (c) shows how the probability of changing the risk percentile for men and women as both age.

For brevity, we here present the results for men and women with only opposite-sex partners; corresponding plots for same-sex individuals are given in the supplementary material, along with the rate by age of opposite-sex partnership formation. These latter distributions are important as STI rates are often higher in groups such as men who have sex with men (MSM) [41], and thus these groups form an important source of infection to the population which models need to capture. Fig 1 shows the parameters that are obtained by matching to Natsal-3 responses. Graphs 1a and 1b show the distributions of the stochastic rate of new sexual partners, *Q*(*P*|*c*), for each percentile risk *P* and for *c* being heterosexual males and females. Fig 1c shows the probability that an individual changes their risk percentile (i.e rate of new partnerships) in a given year—low values (at around 28 years old) correspond to consistent individual behaviour (i.e. people tend to remain within their risk group), which in turn maximises heterogeneity at the population level.

**Fig 2:**
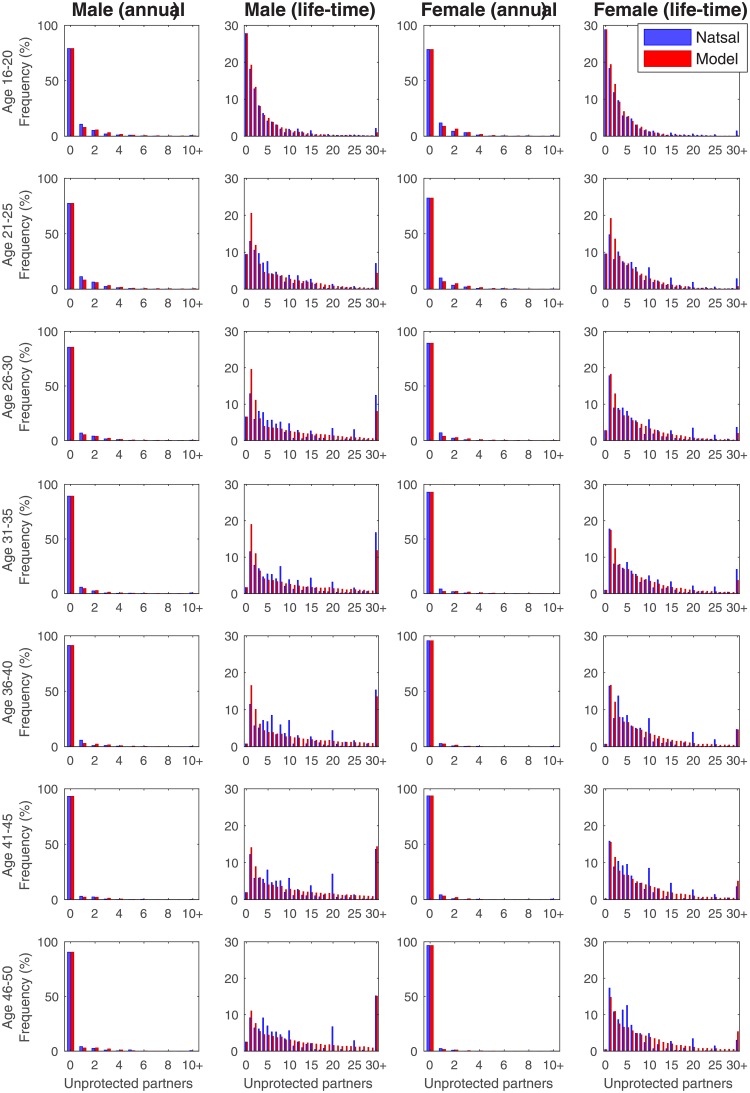
Comparing Natsal-3 data (blue) and the output from model fitting (red). Data separated into men (left two columns) and women (right two columns). Shown are the annual number of sexual partners without condoms (columns 1 and 3) and total lifetime number of partners (columns 2 and 4).

Using these partnership parameters Fig 2 shows a comparison of Natsal-3 survey data (blue) and model simulation (red), displaying both the annual number of new sexual partners without condoms (columns 1 and 3), and the total lifetime number for seven age groups (columns 2 and 4). As expected due to the simple fitting procedure used, the agreement at the annual scale is extremely high; most age-groups rarely form new partnerships and this trend increases with age. The life-time fits are less accurate which may in part reflect participant recall bias or digit preference, with many respondents reporting partnerships that are multiples of five.

### Prevalences from stochastic simulations

**Fig 3:**
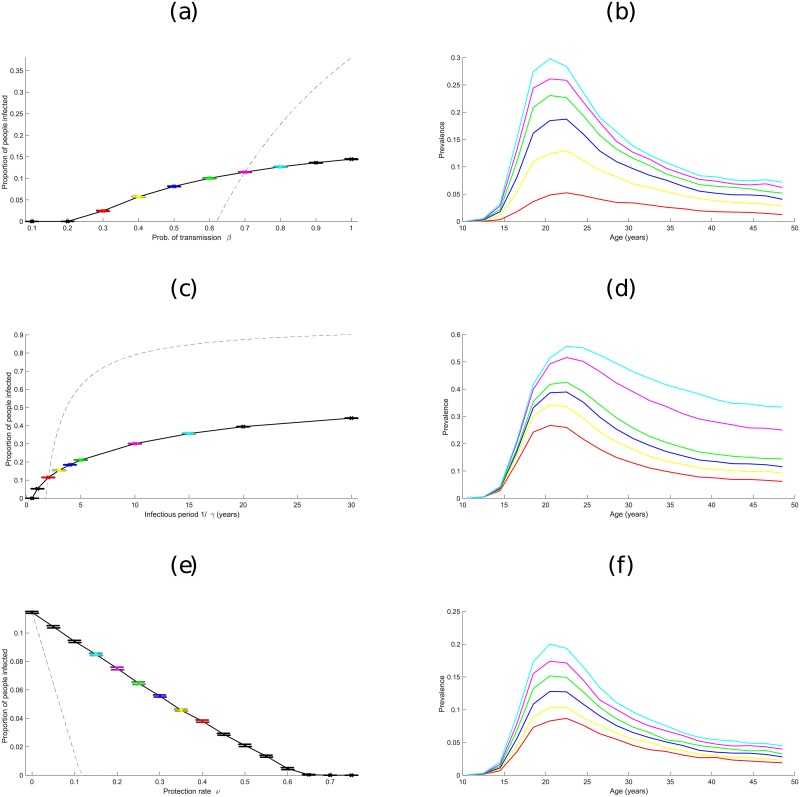
How prevalence of a generic STI varies, both at the population level and by age, at the end of simulations, when varying model parameters. Plots (a), (c) and (e) show the effect of varying transmission probability *β*, recovery rate *γ*, and protection rate *ν* respectively. Dashed lines show the equivalent prevalences using the ODE model given by (3). Confidence intervals are shown by bars. Plots (b), (d) and (f) show prevalence broken down by age at different values of *β*, *γ* and *ν*, with the colour of lines corresponding to particular parameter values in plots (a), (c) and (e). For example, the red line in plot (b) shows prevalence by age when *β* = 0.3 in plot (a), the yellow line corresponds to *β* = 0.4, and so on. (Default values are *β* = 0.7, *γ* = 1 per year and *ν* = 0; these are linked to the deterministic model by assuming $\hat{\beta }\approx 1.65\times \beta$).

Fig 3 displays the effects of varying characteristics of the generic STI on the equilibrium level of infection; specifically, the probability of transmission within a partnership *β*, the average infectious period (1/*γ*) and the proportion of individuals that are protected *ν*. For comparison, we first outline the expected results from a simple (homogeneous) SIS-P (Susceptible—Infected—Susceptible—Protected) model, which are based on coupled ODEs:

$$\begin{array}{c} \begin{array}{ccc} \frac{dS}{dt} & = & \left(1-\nu \right)\mu \;-\;\hat{\beta }SI\;+\;\gamma I-\mu S\\ \frac{dI}{dt} & = & \hat{\beta }SI\;-\;\gamma I-\mu I \end{array}\;\Rightarrow \;I^{*}\;=\;\text{max}(0,\;1-\frac{\nu \hat{\beta }+\gamma +\mu }{\hat{\beta }}) \end{array}$$ (3)

where *γ* is the recovery rate, *ν* is the proportion of individuals that are protected before becoming sexually active, *μ* is the rate that individuals enter or leave the sexually active population (birth rate) and $\hat{\beta }$ is a modified transmission rate which accounts for both partnership formation and transmission within the partnership. We assume that *γ*, *ν* and *μ* take the same values in both the stochastic simulations and the ODE model, but modify $\hat{\beta }$ to obtain the same endemic prevalence of infection at the default parameters. This leads to $\hat{\beta }\approx 1.16$; we therefore use the relationship $\hat{\beta }\approx 1.65\times \beta$ (which gives equal prevalence at the default parameters) to compare the two models (i.e. I* from the ODE model (3) to the equilibrium prevalence in the stochastic simulations) as the parameters are varied (Fig 3). We find that the realistic heterogeneities within the simulation model makes the endemic prevalence of infection far less sensitive to the parameter values compared to the simple ODE model.

We note (although do not show results here) that at the default parameters the ODE model has a much slower growth-rate that the stochastic simulation, despite both having the same equilibrium prevalence; we attribute this to the considerable heterogeneity with the simulations such that a small high-risk group can rapidly transmit infection when it initially invades. In addition, using a more complex ODE model that is homogeneous in terms of risk, but recognises partnership formation [42] does not change the qualitative results of this comparison.

In addition, Fig 3 shows the age-distribution of infection from the simulation model. As might be expected the highest prevalences of infection are in individuals aged 16-25, which is a direct consequence of the Natsal-3 data, as partnership formation rates tend to be highest in this age group [24].

## Discussion

We believe we have generated two main conclusions from our modelling approach, which we now highlight in the discussion. Firstly a relatively simple partnership-formation model can be formulated that captures much of the complexity observed in the Natsal-3 data. In essence, differential partnership formation rates (as shown in Fig 1a and 1b), together with occasion switching to a different risk percentile, is sufficient to capture the heterogeneity distribution of the number of new partners over both short and long time-scales (Fig 2). We would suggest that this model structure could be readily applied to a range of sexually transmitted diseases, although the probability of transmission across a partnership would need to be inferred for each. As an example of this, we focus of human papillomavirus infection which is ubiquitous in the general population. By comparing the results of the simulated heterogeneous population with the predictions of a homogeneous ODE model, we are able to highlight the dramatic impact of the heterogeneities observed in Natsal-3. Most notably, the presence of high-risk (multiple-partner) individuals leads to far less sensitivity to parameter values, even large changes in the transmission and infectious periods leads to relatively small changes in the prevalence of infection. In a similar manner, large proportions (> 60%) of the heterogeneous population need to be successfully protected in order to eliminate the infection, whereas this can be achieved with values above 12% in the homogenous model. These basic results echo more detailed findings in follow-up work which looks at the public-health implications of HPV transmission and vaccination [43].

Previous attempts to simulate STI transmission have either focused on deterministic models, where individuals are divided into subgroups who act identically (e.g. [29, 44–46]), or individual-based microsimulation models that attempt to account for individual-level heterogeneity such as age and sexual preference (e.g. [31, 32]). For the latter, either infection prevalence data [31] or population survey data [32] were incorporated to simulate individual-level behaviour. Other work used STDSIM, an established individual-level stochastic epidemiological model, and input both demographic and prevalence data from different locations to compare epidemic behaviour [47, 48]. With our approach we aimed to use high-resolution data on sexual behaviour in the UK to produce a generic modelling tool that can be adapted to a range of STIs and used to investigate the importance of individual-level heterogeneities in behaviour, in determining the dynamics of the spread of particular STIs. By combining accurate, age-dependent partnership rates with dynamic behaviour switching to capture long-term behaviour in the data, we believe the model framework is a reliable tool for capturing sexual behaviour. It is hoped that future work will build upon the foundation presented here, to add further necessary complexity to better represent the spread of STIs in a realistic way.

Sexual partner change rates determine individuals’ risk for STI acquisition, and the rate of acquiring new partners is correlated with several factors. Here we looked at the effects of age, sex, sexual preference, and partner change rates, known to be important factors in the spread of STIs, using Natsal-3 as our input dataset [28]. As observed in empirical data, for both sexes, sexual activity rises sharply to a peak around 20, before decreasing gradually as age increases, as fewer new partnerships are made (Fig 1a and 1b). Identical trends are observed in men who have sex with men (MSM) and women who have sex with women (WSW) (S1 Fig). The chance of changing new partner rates decreases with age, reaching a minimum around 28, before increasing again. This has the effect of maximising population-level heterogeneity at this age, as individuals tend to retain their risk status. The fitting between Natsal-3 and our model gives tight agreement (Fig 2), validating the use of the distributions for modelling sexual partnership formation in Britain, and likely for countries with similar social structures [14].

We have assumed 100% protection with a condom, so only looked at condomless partnerships. in practice, factors such as delayed application and / or early removal of condoms are likely to reduce condom effectiveness in preventing STI transmission. In addition, condom consistency varies greatly between and within relationships and estimates of condom efficacy are remarkably far from 100%. An estimated 56% on Natsal-3 participants (aged 16-74 yrs, although 90% were under 45 years old) reported that they had used condoms on the first occasion with their most recent partner (of those who reported new partner(s) within the past year. A recent study showed that, where condoms are used at first sex with a partner, then use quickly wanes, typically within 21 days of first sex together (in most cases because other forms of long-acting reversal contraception are used instead, e.g. the pill) [49]. Further work would look at the impact of these heterogeneities on infection transmission and control.

Adding a generic STI with simple SIS-P dynamics to the mixing model, results in disease prevalences that correlate with the new partnership formation rates from Fig 1; namely, age groups with higher partnership formation rates have increased prevalences of infection (inset plots of Fig 3). High population prevalences are associated with a higher probability of transmitting infection, longer infectious period and lower level of population protection, as is expected intuitively. Interestingly, while increasing *β* and decreasing *ν* increased prevalence linearly across all age groups, increasing the infectious period 1/*γ* broadened the age range of those infected, such that older age groups were increasingly infected. This is a result of people beginning to form sexual partnerships at the same times, but taking longer to recover from infection and thus remaining infected until later in life.

Comparing the basic ODE formulation of the SIS-P model (Eq (3)), with the individual-based stochastic simulations, reveals the differences that heterogeneity in partner selection can make to prevalence within a population. Although default parameters were suitably chosen to give identical baseline prevalences, the ODE approach is far more sensitive to changes in the three model parameters. This heterogeneity can be particularly important when assessing control through vaccination; in the ODE model the infection can be eradicated by vaccinating about 13% of the population, whereas in the simulation in excess of 50% is required—although interestingly we still obtain a linear relationship.

A necessarily simple epidemiological approach has been taken here, to exemplify the uses of the partnership formation rates derived from Natsal-3, and to compare the ODE and stochastic model approaches. SIS-P dynamics were selected to reduce the number of parameters necessary, while allowing infection to persist in the population for large regions of parameter space. More complex dynamics such as SIRS (Susceptible—Infected—Recovered—Susceptible), incorporating a temporary immune period, are also possible using the modelling framework [43]; further follow-up work should likewise alter infection dynamics as necessary so that they represent the realistic acquisition and clearing of the STI of interest. The ODE model presented here had homogeneous individuals for analytical convenience and to illustrate the impact of heterogeneity on equilibrium behaviour; in reality, in an ODE model approach factors such as age, sex, sexual activity and partner change rates should be included, similar to the stochastic simulations shown here [31]. Additional factors such as alcohol consumption [50], smoking [51] and socioeconomic status [19] are observed to have an influence on partnership formation rates, and including these in future models will also increase the robustness of results. While it would in theory be possible to generate an ODE version of the underlying heterogeneous dynamics that form the simulation model, this is impractical do to the exceedingly high dimension of the system, which requires both age and risk structure, as well as epidemiological status. We therefore use a stochastic individual based model which allows us to more carefully follow the temporal dynamics of individuals.

Concurrency is a potentially important source of infection transmission. Theoretical studies have shown their impact on epidemic size (e.g. [52], and along with high-risk behaviour, an important factor in the establishment of infection [25]. In particular, it is thought that understanding of concurrency is key in controlling epidemic sizes of HIV [53], and correlations have subsequently been found between concurrency and HIV levels [54]. The accurate modelling of concurrency will provide more robust results about STI transmission rates [27, 55]. In particular, one such aim would be correctly modelling partnerships which are not behaviourally concurrent (as individuals may be behaviourally serially monogamous) but biologically concurrent, if the gap in time between partners is less than the infectious period of an STI [56]. In the work shown here, our model does include the ‘new partnership’ caused by any form of concurrency (but not the impact this might have within the ‘stable’ partnership). However, recently published work motivated by this concern clearly shows that in a well-fitted model the general effect of concurrency is limited [57]. On a related topic, future work should focus on the role of partnership duration and casual partnerships on STI transmission; an analysis on Natsal-2 data showed that a substantial minority of partnerships in the UK were casual [58], and failing to account for this could lead to underestimating STI risk in the population.

As a simplification in the stochastic simulations, individuals were chosen at random to be protected against infection. If we take protection to mean vaccination here, there is evidence at the individual level of a relationship between vaccination and risk behaviour [59, 60]. When this risk is highly clustered to particular groups, for example for hepatitis-B, then the ability to target these groups can be critical to the decision to vaccinate [61]. To test this effect in the model, additional stochastic simulations were run where the most risk-prone individuals were protected (rather than selecting individuals at random) when they entered the population. it was found that there was no difference in the overall prevalence of infection when protecting in this way. This is because both low- and high-risk individuals were able to change their risk status throughout their lifetime (mirroring changing sexual behaviours over time), and so the risk status upon entering the population at age 10 was not correlated with the lifetime number of partners. To expand upon the simple framework presented here, one should assess the importance of risk groups and their relative likelihood of vaccination, the mixing between the groups, and the effectiveness of vaccination given the relative coverage in different groups.

## Conclusion

What has been presented here is a general framework for allowing individuals to partner other individuals at realistic rates given one’s sex, sexual preference and age. It is hoped that the partnership formation rates described will be a reliable modelling tool in the future (much in the same way as [14] for describing general contact patterns) for simulating the spread of STIs in countries like Britain, and assessing the potential impact that prevention measures such as vaccination can have.

## Supporting information

S1 Fig How partnership formation rates for MSM and WSW change with age. Plot (a) shows the partnership formation rate distributions for MSMs, and plot (b) shows how the probability of picking a female partner changes with age among MSM. Likewise, plot (c) shows the partnership formation rate distributions for WSWs, and plot (d) shows how the probability of picking a male partner changes with age. We define men who have sex with men (MSM) as males who have had one or more same-sex partners in the past, or who will have one in the future. Note that some MSM will have one or more opposite-sex partners while others will not. (Similarly for women who have sex with women (WSW).) The partnership formation rates for MSM and WSW of different ages are shown (analogous to Fig 1), as well as probabilities of picking female (male for WSW) partners with age.
(EPS)

S1 Table Mixing matrices. The attached Excel file contains sheets with all the data needed to recreate the sexual mixing patterns presented in this article. The sheets show, in order: (1) female age mixing matrix, (2) male age mixing matrix, (3) change of risk group with age, (4) female partnership formation rate percentiles with age if virgin; (5) female partnership formation rate percentiles with age without a condom (no longer virgin); (6) male partnership formation rate percentiles with age if virgin; (7) male partnership formation rate percentiles with age without a condom (no longer virgin); (8) MSM partnership formation rate percentiles with age; (9) WSW partnership formation rate percentiles with age.
(CSV)

S1 File Code for statistical fitting. Alongside the anonymised dataset, the Natsal-3 codebook, giving details of all variables in the dataset, is also available to academic researchers from the UK Data Service, https://discover.ukdataservice.ac.uk/; SN: 7799; persistent identifier: http://doi.org/10.5255/UKDA-SN-779-2. Code is provided to analyse the Natsal-3 data as a Matlab file [62]. The data (as a txt file) and the code should be placed in the same directory; the code should be called as a Matlab script that will read in the data, fit the annual pattern of new partners, calculate the age-structured mixing and infer the changes in risk-percentile with age, it will also generate the set of figures as seen in the paper.
(M) Click here for additional data file.
