# Correction: Capturing sexual contact patterns in modelling the spread of sexually transmitted infections: Evidence using Natsal-3

**DOI:** 10.1371/journal.pone.0315237

**Published:** 2024-12-04

**Authors:** Samik Datta, Catherine H. Mercer, Matt J. Keeling

S1 Table is uploaded incorrectly. Please view the correct S1 Table below.

## Supporting information

S1 Table Mixing matrices. The attached Excel file contains sheets with all the data needed to recreate the sexual mixing patterns presented in this article. The sheets show, in order: (1) female age mixing matrix, (2) male age mixing matrix, (3) female partnership formation rate percentiles with age if virgin, (4) female partnership formation rate percentiles with age without condom (no longer virgin), (5) male partnership formation rate percentiles with age if virgin, (6) male partnership formation rate percentiles with age without condom (no longer virgin, (7) MSM partnership formation rate percentiles with age, (8) MSM probability of female partner with age.
(XLSX)
